# Negative Mood and Obsessive-Compulsive Related Clinical Constructs: An Examination of Underlying Factors

**DOI:** 10.3389/fpsyg.2017.01570

**Published:** 2017-09-14

**Authors:** Gary I. Britton, Graham C. L. Davey

**Affiliations:** ^1^School of Human and Social Sciences, University of West London Brentford, United Kingdom; ^2^School of Psychology, University of Sussex Brighton, United Kingdom

**Keywords:** obsessive-compulsive disorder, inflated responsibility, intolerance of uncertainty, not just right experiences, checking stop rules, negative mood

## Abstract

Emerging evidence suggests that many of the clinical constructs used to help understand and explain obsessive-compulsive (OC) symptoms, and negative mood, may be causally interrelated. One approach to understanding this interrelatedness is a motivational systems approach. This approach suggests that rather than considering clinical constructs and negative affect as separable entities, they are all features of an integrated threat management system, and as such are highly coordinated and interdependent. The aim of the present study was to examine if clinical constructs related to OC symptoms and negative mood are best treated as separable or, alternatively, if these clinical constructs and negative mood are best seen as indicators of an underlying superordinate variable, as would be predicted by a motivational systems approach. A sample of 370 student participants completed measures of mood and the clinical constructs of inflated responsibility, intolerance of uncertainty, not just right experiences, and checking stop rules. An exploratory factor analysis suggested two plausible factor structures, one where all construct items and negative mood items loaded onto one underlying superordinate variable, and a second structure comprising of five factors, where each item loaded onto a factor representative of what the item was originally intended to measure. A confirmatory factor analysis showed that the five factor model was preferential to the one factor model, suggesting the four constructs and negative mood are best conceptualized as separate variables. Given the predictions of a motivational systems approach were not supported in the current study, other possible explanations for the causal interrelatedness between clinical constructs and negative mood are discussed.

## Introduction

A number of clinical constructs have been identified and causally linked to obsessive-compulsive (OC) symptoms (Davey, 2003). These constructs aim to capture the beliefs, attitudes, and thought patterns associated with OC symptoms, and examples of such constructs include intolerance of uncertainty (IU; Beech and Liddell, 1974) and inflated responsibility (Salkovskis, 1985). In addition to the aforementioned clinical constructs, negative mood has also been casually linked to OC symptoms. For example, Salkovskis and Freeston (2001) proposed that negative mood may increase the occurrence of intrusive thoughts, increase the accessibility of negative assumptions, increase the likelihood of inadequate appraisals and decrease the efficacy of dismissal, suppression, and other neutralizing strategies. Whilst on occasion two or more of these clinical constructs maybe connected together in a causal model (see, e.g., Lind and Boschen, 2009) more often these constructs are treated as separable and as having separable causal effects on OC symptoms. The aim of the present study is to examine if clinical constructs related to OC symptoms and negative mood are best treated as separable or, alternatively, if these clinical constructs and negative mood are best seen as indicators of an underlying superordinate variable or variables.

Consistent with the idea that constructs are separable, constructs are usually measured using separate inventories or subscales (see, e.g., Obsessive Compulsive Cognitions Working Group [OCCWG], 1997) and constructs have been manipulated independently of one another to examine their effect on OC symptoms (e.g., Ladouceur et al., 1995). Most theories are silent on any potential relationship between constructs or explicitly state they believe constructs to be separable (e.g., Summerfeldt, 2004, 2007) and, when constructs are examined together within a single study, they are usually placed head to head against one another to see which construct “best” predicts OC symptoms (e.g., Steketee et al., 1998) – an approach which emphasizes the supposed separable nature of the constructs and the differences, as opposed to the similarities, between them. This approach is often taken in studies even when the relevant constructs have been shown to be at least moderately correlated with one another within the same study (e.g., Steketee et al., 1998).

However, emerging research suggests that constructs may interact to increase OC symptoms and that constructs themselves may be causally interrelated. For example, using a mediation model, Lind and Boschen (2009) found that the relationship between inflated responsibility and checking was fully mediated by IU. In a series of three experiments that explored the causal relationships between inflated responsibility, IU and negative mood, Britton and Davey (2014) found that all three constructs were causally interrelated. Similarly, Dash and Davey (2012) found that manipulating negative mood casually affected the deployment of as-many-as-can (AMAC) stop rules, whilst Britton (2011, unpublished) found that manipulating inflated responsibility increases the intensity of “not just right experiences” (NJRE).

Britton and Davey (2014) interpreted their results within a motivational systems approach. In this approach emotions such as anxiety are seen as features of a ‘precautionary system’ that simultaneously alerts the individual to challenges and threats to goals, and coordinates cognitive and behavioral reactions in order that the individual can respond more effectively to these challenges and threats. Individual threat management systems such as this will be characterized by a functional coherence in which perceptual, affective, cognitive, and behavioral processes work together to reduce the fitness costs of potential threats (e.g., Frijda, 1986; Keltner et al., 2006). As perceptual, affective, cognitive, and behavioral elements are all part of an integrated evolved functional system, we would expect these elements to be highly coordinated and interdependent, with the affective experience being an emerging property of the activation of the various functional elements in the system (Kenrick and Shiota, 2008; Neuberg et al., 2011). Britton and Davey (2014) argued that if disorders such as OC disorder (OCD) are fundamentally derived from anxiety as an adaptive emotion then one implication of the motivational systems view is that emotional, cognitive, and behavioral elements characteristic of anxiety should be coordinated and interdependent within the threat management system relevant to anxiety, and the integrated nature of the relationships between negative mood and constructs such as inflated responsibility and IU are supportive of such a view. Rather than one set of factors (e.g., constructs) being causes of a different set of factors (e.g., affect), they are all integrated components of an anxiety precautionary system that promotes a ‘cascade’ of relevant perceptions, cognitions, behaviors, and affective experience conducive to solving the adaptive problem (Kenrick et al., 2010).

The primary purpose of this paper is to further examine the underlying relationship between four constructs related to OCD and negative mood. Specifically, the primary aim of the current study was to examine if these clinical constructs and negative mood are separable or if they are indicators of a single superordinate variable, as would be suggested by a motivational system approach, or, alternatively, if they are indicative of a different number of underlying variables. Due to the large number of clinical constructs linked to OCD within the literature, the authors’ chose to include only those constructs which have been shown to be casually related to each other and/or with negative mood within the literature.

In addition to negative mood, the clinical constructs IU and inflated responsibility were measured in the current study as evidence suggests they have bidirectional relationships with both negative mood and with each other (Britton and Davey, 2014). *IU* is defined as a “dispositional characteristic that arises from a set of negative beliefs about uncertainty and its connotations and consequences” (Birrell et al., 2011, p. 1200) and is underpinned by appraisals such as ‘uncertainty is dangerous,’ ‘uncertainty is intolerable,’ and ‘I can’t deal with uncertainty’ (Koerner and Dugas, 2006). *Inflated responsibility* is defined as the belief that one has the power to bring about or prevent subjectively crucial negative outcomes (Salkovskis, 1985; Rachman, 1998).

Two further constructs were also measured in the current study as evidence suggests that they are causally facilitated by negative mood, IU or inflated responsibility (Britton, 2011, unpublished; Dash and Davey, 2012) and therefore these constructs may potentially be coordinated and interdependent within any relevant threat management system. *NJREs* can be defined as, “the subjective sense that something isn’t just as it should be,” an unsettled feeling due to something in the individual or in the world around them not being right (Coles et al., 2003). The final construct focused on in this paper is *“AMAC” goal-directed stop rules* for checking. Stop rules can be best explained by linking them to task motivation. Broadly, two specific types of task motivation have been proposed, performance focused motivation and task focused motivation (Vaughn et al., 2006). A performance motivated individual who engages in a task will be focused on meeting a certain standard or criteria whilst engaged in that task. The person motivated in this way is likely to continue with the task until they have met their given standard or criteria for that task (e.g., Hirt et al., 1996). In contrast, a task motivated individual who engages in a task will do so without concern about evaluation or without any particular performance standards for the task. A person using AMAC stop rules whilst engaged in a task (such as checking or worrying) is analogous to someone using performance focused motivation, the individual’s AMAC stop rule for that task will encourage them to continue with the task until they are sure they have met whatever their specific criteria or standard was for that task.

It is of note that all four constructs measured in the current study have been shown to have a causal effect on OC symptoms (Ladouceur et al., 1995; Coles et al., 2005; MacDonald and Davey, 2005; Toffolo et al., 2013) and each have also been linked to anxiety related symptoms (Ladouceur et al., 2000; Coles et al., 2003; Startup and Davey, 2003) making an exploration of the relatedness of these constructs also of relevance to anxiety disorders. In summary, the primary aim of the current study is to explore the underlying relationships between negative mood and four OC symptom related constructs which recent evidence suggest are causally interrelated. A threat management system approach would suggest that each construct and negative mood would be best depicted as an indicator of a single superordinate variable whilst, if constructs and negative mood are separable, we would predict that a five factor model would be the best depiction of these relationships, with each of the four constructs and negative mood, respectively, represented by a single factor. Plausible arguments could be made for other factor solutions. For example, Summerfeldt’s (2004; 2007) model of OCD proposes two core, continuous, orthogonal dimensions to explain the motivational processes important to the development and maintenance of OCD: harm avoidance (as characterized by inflated responsibility) and incompleteness (as characterized by NJRE). This model would suggest that NJRE and inflated responsibility should be represented by two separate factors. However, it would be difficult to predict based on this model if negative mood, for example, should load onto either of these two factors or a separate factor.

In order to explore the factor structure underlying the four constructs and negative mood an exploratory factor analysis was first carried out in order to ascertain possible factor structures underlying the relationships between the four measured constructs and negative mood. Any emerging plausible models based on the findings of this exploratory factor analysis were then directly compared with one another using confirmatory factor analysis.

## Materials and Methods

### Participants

A questionnaire booklet was completed by a student sample of 370 participants (male: 74; female: 296). Ages ranged from 17 to 74 years (*M* = 27.38, *SD* = 11.96). 48.3% of the sample in the current study consisted of psychology undergraduates at the University of Sussex who received partial fulfillment of a course requirement by taking part in the study. The reminder of the sample represent other students, university staff, and university visitors who volunteered to fill in the questionnaire after being initially approached by the researcher. This latter groups of participants received the gratitude of the researcher for participation but were not financially rewarded.

### Procedure

Participants were provided with questionnaire-batteries, with every second questionnaire package reverse ordered. Participants were asked to supply some very basic demographic information and to provide informed consent before completing the questionnaire.

This study was carried out in accordance with the recommendations of British Psychological Society with written informed consent from all subjects. All subjects gave written informed consent in accordance with the Declaration of Helsinki. The protocol was approved by the ethics committee at the University of Sussex.

### Measures

Intolerance of uncertainty was measured using the Intolerance of Uncertainty Scale (IUS, Freeston et al., 1994), which was designed to measure an individual’s IU, particularly the ideas that uncertainty is unacceptable, reflects badly on a person, leads to frustration and stress, and leads to the inability to take action. The IUS has demonstrated excellent internal consistency (α = 0.94), good test–retest reliability (*r* = 0.78), and convergent and divergent validity (Buhr and Dugas, 2002). The IUS had excellent internal consistency in the current study (α = 0.95).

Not just right experiences were measured using the Not Just Right Experiences-Questionnaire Revised (NJRE-QR, Coles et al., 2003) which is composed of 19 items. The first 10 items measure how often NJRE occur. The next two items (items 11 and 12) ask respondents to indicate which NJRE occurred most recently and when it last occurred (past few hours to past month). The last 7 items in the questionnaire measure the intensity of NJRE. The NJRE-QR produces two total scores, NJRE occurrence (composite score of NJRE-QR items 1–10) and NJRE intensity (composite score of NJRE-QR items 13–19). Coles et al. (2003) found good internal consistency (α = 0.79) for the 10 NJRE occurrence items, and all 19 items showed good convergent and discriminant validity, evident in stronger correlations with OCD symptoms than with depressive symptoms, trait anxiety, social anxiety or worry. In the current sample the NJRE occurrence subscale showed acceptable internal consistency (α = 0.74) whilst the NJRE intensity scale showed excellent internal consistency (α = 0.94).

Negative mood was measured using the Positive and Negative Affect Schedule (PANAS, Watson et al., 1988) which consists of two 10-item mood scales. The first is a measure of positive affect and lists 10 “positive” emotions and the second is a measure of negative affect and lists 10 “negative” emotions. Watson et al. (1988) report that both scales have good internal consistency (reliability of the positive affect scale ranged from α = 0.86 to α = 0.90, the negative affect scale from α = 0.84 to α = 0.87). The construct validity of the scale has been supported (see Crawford and Henry, 2004). In the current sample both the positive affect scale (α = 0.87) and the negative affect scale (α = 0.88) showed good internal consistency.

Inflated responsibility was measured using the Responsibility Attitude Scale (RAS; Salkovskis et al., 2000), a 26-item questionnaire that measures general beliefs related to inflated responsibility. The internal consistency of the scale is excellent and test–retest reliability is also excellent (*r* = 0.94, Salkovskis et al., 2000). Several studies attest to the measures convergent validity (Salkovskis et al., 2000; Yorulmaz et al., 2002). The RAS had excellent internal consistency in the current study (α = 0.92).

As-many-as-can checking stop rules were measured using the Checking Stop Rule Questionnaire (CSRQ, Britton, 2011, unpublished), a 20-item questionnaire where 10 items measure endorsement of AMAC stop rules and where 10 measure endorsement of “feel like continuing” (FLC) stop rules. Britton (2011, unpublished) reported that two factors underlie the CSRQ, the first measuring AMAC stop rules and the second FLC stop rules and that both of these factors are reliable (reliability for AMAC subscale was α = 0.91, reliability for the FLC subscale was α = 0.88). The same study found that the CSRQ’s two subscales correlate in expected directions with other relevant constructs providing evidence of the CSRQ’s validity (Britton, 2011, unpublished). In the present study the AMAC subscale had excellent internal consistency (α = 0.91) whilst the FLC subscale had very good internal consistency (α = 0.89).

## Results

### Missing Data

There was very little missing data in the sample; overall 99.12% of the total number of questions were answered across the sample. Therefore, any missing data was imputed by adding the mean of the relevant question.

### Preliminary Analysis

A preliminary analysis was conducted to examine the Pearson’s correlation between the total scores (or relevant subscale scores) on the questionnaire measures of the four constructs and negative mood. IU, inflated responsibility, negative mood, AMAC stop rule use, and NJRE occurrence and intensity were all significantly correlated, with correlations ranging from medium to large in terms of size (correlations ranging from 0.36 to 0.69, see **Table 1**). From this preliminary analysis it is realistic to assume that IU, inflated responsibility, negative mood, AMAC stop rule use, NJRE occurrence, and NJRE intensity all overlap and possibly reflect some underlying superordinate variable.

**Table 1:** Pearson correlation coefficients between the total scores on the four clinical constructs and negative mood.

	IU	Inflated responsibility	Negative mood	NJRE occurrence	NJRE intensity	AMAC
IU	–	0.59^∗^	0.56^∗^	0.48^∗^	0.44^∗^	0.48^∗^
Inflated responsibility		–	0.42^∗^	0.36^∗^	0.36^∗^	0.48^∗^
Negative mood			–	0.37^∗^	0.37^∗^	0.40^∗^
NJRE occurrence				–	0.69^∗^	0.43^∗^
NJRE intensity					–	0.44^∗^
AMAC						–

^∗^*p < 0.001. Two-tailed significance reported*.

### Analytic Strategy and Treatment of Categorical Data

In order to explore if the constructs and negative mood are separable or if they are best seen as indicators of one (or more) superordinate variables, a two-stage approach was taken. Firstly, an exploratory factor analysis was conducted to provide an indication of how many factors may underlie the data set. Secondly, plausible factor structures (as suggest by the exploratory factor analysis) were compared directly using confirmatory factor analysis.

Items entered into a factor analysis should generally be continuous as opposed to categorical (Kline, 2005). Within the current study, 10 items were measured on scales with less than five levels. These 10 items are the first 10 items of the NJRE-QR. Each of these items ask the participant to state if they have experienced a specific NJRE within the past month (e.g., I have had the sensation after getting dressed that parts of my clothes tags, collars, pant legs, etc., didn’t feel just right) and participants are simply asked to offer a yes or no response. Kline (2005) suggests one way to overcome the problem of categorical items in factor analysis is to parcel items together, that is to create one or more total scores (linear composites) across a set of two or more items. These parcels can then be treated as continuous indicators. It was decided to therefore make two composite variables (both of which would have a range of possible scores from 0 to 5). The response to NJRE items 1, 2, 3, 4, and 5 were combined to make a composite score, NJRE occurrence 1. In support of the combination of all of these items into one score, all of the individual items were significantly positively correlated with each other (all correlations significant at *p* < 0.001). The responses to NJRE items 6, 7, 8, 9, and 10 were combined to make a second composite score, NJRE occurrence 2. In support of the combination of all of these items into one score, all of the individual items were significantly positively correlated with each other (all correlations significant at *p* < 0.01). With these 10 variables transformed into two composite scores, all variables in the data set were now measured on a scale with at least five levels.

### Exploratory Factor Analysis

An exploratory factor analysis was performed on the four constructs and negative mood to explore the factor structure underlying these variables. All of the 26 items measuring inflated responsibility (the RAS), the 27 items measuring IU (the IUS), the 10 items measuring AMAC stop rule use (from the CSRQ), and the 10 items measuring negative mood (from the PANAS) were examined in the analysis. In addition, the two composite NJRE occurrence variables described in the previous section and the items in the NJRE-QR measured on separate 7-point Likert scales (items 13–19) were also examined in the analysis (giving a total of 82 items).

Communalities ranged from 0.50 to 0.84. Fifteen components had eigenvalues over 1: 23.29, 5.02, 4.59, 3.37, 3.15, 1.97, 1.70, 1.54, 1.36, 1.25, 1.20, 1.13, 1.12, 1.07, and 1.01. The scree plot was used to determine the optimum number of factors (as recommended by, e.g., Catell, 1966; Field, 2009). The scree plot strongly indicated a one or five factor solution over alternative factor solutions (e.g., a two factor solution or three factor solution) and so these two possible factor structures were further explored.

Firstly, a factor analysis was run extracting one factor. This solution explained 23.29% of the variance. The internal consistency for this scale was excellent (α = 0.96). Examination of the factor loadings showed that while most of the items had moderate loadings (0.40 or above, Field, 2009) on the one emergent factor, 9 items did not. Of the items which did not load moderately onto the emergent factor, 7 were from the RAS, 1 from the CSRQ, and 1 from the PANAS.

Secondly, a factor analysis was run extracting five factors with varimax rotation (a varimax rotation was used to aid with interpretation of the emergent factors, however, it is of note that an oblique rotation was also run which produced a nearly identical factor solution to the varimax rotation. The results of the oblique rotation are therefore not reported). After rotation the five emergent factors had eigenvalues of: 12.28, 8.55, 6.65, 6.23, and 5.61. This solution explained 47.94% of the variance. Looking at the rotated component matrix the resulting scale produced five reliable subscales each separately measuring each of the original five constructs; IU, inflated responsibility, negative mood, NJRE, and AMAC stop rules (see **Table 2** for internal consistency, means and standard deviations on the five scales and correlations between factors). Of note is the fact that *all* of the items thought to measure a particular construct loaded most strongly onto the factor thought to represent that construct.

**Table 2:** Descriptive statistics for the five constructs and correlations between factors (*n* = 370).

	Internal consistency	Mean (SD)	2	3	4	5
(1) AMAC	α = 0.91	2.46 (0.90)	0.48^∗^	0.48^∗^	0.44^∗^	0.40^∗^
(2) Inflated responsibility	α = 0.92	3.60 (0.91)		0.60^∗^	0.37^∗^	0.41^∗^
(3) IU	α = 0.95	1.98 (0.71)			0.47^∗^	0.55^∗^
(4) NJRE	α = 0.91	4.72 (3.64)				0.39^∗^
(5) negative mood	α = 0.89	2.59 (0.81)				

^∗^*p < 0.001. Two-tailed significance reported*.

### Confirmatory Factor Analysis

A confirmatory factor analysis was performed to directly compare the five factor model and the one factor model.

The one factor model was specified so that all items loaded directly onto one factor. In the five factor model items were specified to load onto only one of five factors according to the pattern indicated by the five factor solution (i.e., all IU items loading only onto factor 1, or the IU factor, all RAS items loading only onto factor 2, or the inflated responsibility factor, all negative mood PANAS items load only onto factor 3, or the negative mood factor, all NJRE-QR factors loading only onto factor 4, or the NJRE factor, all AMAC CSRQ items loading only onto factor 5, or the AMAC factor). In the model all five factors were specified to correlate with one another (as is suggested in **Table 2**).

This analysis showed the five factor model is a significantly better fit than the one factor model, Δχ^2^ (10) = 4840, *p* < 0.001. The five factor solution is therefore preferred over the one factor solution.

It should be noted that neither the one factor nor the five factor model were an especially good fit to the data using many conventional fit indices, although observation of these indices support the findings of the chi-square test, suggesting that the five factor model is a better fit to the data than the one factor model. The reason for such poor fit, in relation to both models, is because a large number of significant pathways were not specified in the models as doing so would have compromised the point of the analysis: to test the relative fit of a one factor vs. five factor model. A list of the significant pathways not added to the models by type are: correlations between error terms, correlations between items, correlations between error terms and items (five factor model only), correlations between error terms and latent variables (five factor model only), and correlations between items and latent variables (five factor model only). Values for selected fit indices for the one factor model are: CFI = 0.51, RMR = 0.22, RMSEA = 0.08 with 90% confidence interval 0.08–0.09. Values for selected fit indices for the five factor model are: CFI = 0.79, RMR = 0.12, RMSEA = 0.06 with 90% confidence interval 0.05–0.06.

## Discussion

The analyses reported in this paper demonstrate that inflated responsibility, IU, NJRE, AMAC stop rules, and negative mood are best seen as five separate variables rather than as indicators of an underlying superordinate variable or variables. The exploratory factor analysis suggested two plausible factor structures, one where all construct items and negative mood items loaded onto one underlying superordinate variable, and a second structure comprising of five factors, where each item loaded onto a factor representative of what the item was originally intended to measure (i.e., all IU items loading only onto factor 1, or the IU factor, all RAS items loading only onto factor 2, or the inflated responsibility factor, etc.). A confirmatory factor analysis showed that the five factor model was preferential to the one factor model, suggesting the four constructs and negative mood are best conceptualized as separate variables.

The results of the present study are therefore not supportive of a motivational systems approach in relation to explaining the relationships between constructs related to OC symptoms and negative mood (Britton and Davey, 2014). Such an approach would suggest that, as the constructs measured and negative mood are highly coordinated and interdependent within the relevant threat management system, they should all load onto one superordinate variable representative of that threat management system. Rather, the results of the present study suggest the four constructs and negative mood are separable and therefore support the fact that each of the clinical constructs are generally discussed, measured and manipulated separately from each other within the OCD literature. The results are also supportive of those theories which suggest that the constructs are separable, for example, Summerfeldt’s (2004, 2007) model of OCD which proposes two dimensions to explain the motivational processes important to the development and maintenance of OCD: harm avoidance (as characterized by inflated responsibility) and incompleteness (as characterized by NJRE). In addition, the results of the present study are supportive of those theories which suggest that clinical constructs and negative mood are separable, for example, Salkovskis et al.’s (2000) model which suggests that inflated responsibility and negative mood are separate but causally interrelated variables which both increase the occurrence of intrusive thoughts. Cognitive treatments for OCD and anxiety disorders are often based on addressing the types of clinical constructs measured in the current paper (e.g., inflated responsibility, Kohlenberg and Vandenberghe, 2007; IU, Dugas and Ladouceur, 2000). The results of the present study support the idea that it possible to separately address the clinical constructs measured in the current study in the treatment of OCD and anxiety disorders.

The finding that the constructs measured and negative mood seem to be separable rather than indicators of a core underlying variable raises a question: how do we explain bidirectional causal relationships between negative mood with constructs related to OC symptoms, and the bidirectional relationship between constructs themselves (e.g., Britton and Davey, 2014), if they are not indictors of one superordinate variable?

One possibility is that although constructs related to OC symptoms and negative mood are unique and separable entities they are entities which to some extent overlap with one another, as indicated by the fact that all four constructs measured in the current study and negative mood appear to correlate moderately with one another. As such, the manipulation of one construct or negative mood will have a causal influence on other constructs measured (or negative mood), due to the overlapping relationship between the relevant constructs and negative mood.

Another possibility is that that while constructs related to OC symptoms and negative mood represent unique and separable entities, they are all connected to a third variable which mediates the relationship between them. One potential candidate for mediating the relationship between inflated responsibility, IU and negative mood (Britton and Davey, 2014) is that all three constructs affect information processing style, and in particular trigger systematic processing of information. Systematic processing is a bottom-up, data-driven, and comprehensively analytic style in which perceivers access and scrutinize all informational input for its relevance and importance to their judgment, and integrate all information in forming their judgment (Chaiken et al., 1989). Negative mood has been shown experimentally to facilitate systematic processing (Batra and Stayman, 1990; Tiedens and Linton, 2001; Ambady and Gray, 2002) as have a range of situational and dispositional factors including responsibility, accountability, desire for control, personal relevance, and task importance (Sorrentino et al., 1988; Chaiken et al., 1989; Batra and Stayman, 1990; Maheswaran and Chaiken, 1991; Lee et al., 1999; Tiedens and Linton, 2001; Ambady and Gray, 2002) and many of these factors are likely to be enhanced by feelings of inflated responsibility and IU. For example, feelings of IU have been linked to increases in desire for control (Krohne, 1993) whilst increases in inflated responsibility are likely to lead to an increased sense of task importance (Salkovskis, 1985). Negative mood in particular provides information that characterizes a situation as problematic and fosters the spontaneous adoption of a systematic, detail-oriented, bottom-up processing style (Schwarz, 1990). Increasing feelings of responsibility will also signal a situation as problematic and foster systematic processing (Chaiken et al., 1989) – but only if the outcomes for which the individual feels responsible are appraised as having potentially harmful or threatening outcomes, a characteristic typical of the OC explanatory construct of inflated responsibility (Salkovskis, 1985). An increase in IU will increase the desire for control (Krohne, 1993) and this in turn will also foster systematic processing (Maheswaran and Chaiken, 1991). Thus, inflated responsibility, IU, and negative mood are all factors that potentially have an integrated relationship with a specific information processing style which may explain the bidirectional causal relationships between them. Activation of that common processing style through increases in inflated responsibility, IU or negative mood, respectively, is likely to lead to an increase in scores on the other two constructs, as all three constructs are associated with increases in systematic processing. It is also of note that those individuals with OCD and OC tendencies use a more controlled information processing style, even under conditions that non-OCD participants use a speed-oriented information processing style (Soref et al., 2008; Kalanthroff et al., 2014).

Whilst the four constructs measured in the current study and negative mood appear to be separate variables, the fact that one factor explained a good deal of shared variance between the four constructs and negative mood in the exploratory factor analysis is of note. Whilst this factor may have simply been artifact of shared method variance, it is possible it maybe representative of a core genetic/biological deficit underlying OC symptoms (e.g., Maia et al., 2008) or anxiety related problems more generally (e.g., Norrholm and Ressler, 2009).

Finally, the present study has a number of limitations. First, given a primarily student sample was used in the present study, it is not clear how generalisable the conclusions of this study are to a clinical population. It should be noted that taxometric studies have suggested that OC related symptoms are generally best considered as dimensional rather than categorical (e.g., Haslam et al., 2005) and many cognitive models of OC symptoms follow a dimensional model (e.g., Frost and Steketee, 2002) supporting the appropriateness of studying OC related phenomena in student samples. However, the validity of the results presented in this paper would be strengthened if they were replicated within a clinical sample. Second, the present study measured only four constructs, and negative mood, and as such did not measure a number of other constructs that have been linked to OC symptoms (e.g., thought-action fusion, Rachman, 1993). Whilst this was done as we wished to include only those constructs which have been connected causally through experimental manipulation, it should be noted that the inclusion of other constructs may have led to a different factor structure emerging in the current study. A related limitation of the present study is that OC symptoms and anxiety symptoms which have been linked to the constructs measured in the current study (e.g., worry) were also not measured. Again, the inclusion of OC symptoms and symptoms related to other disorders may have led to a different factor structure emerging in the current study. Finally, only self-report measures were used in the current study. Whilst noting the limitations associated with self-report measures, the authors’ would comment that the constructs measured in the current study are almost exclusively measured by self-report in the wider OC literature, and so measurement of these constructs in this study is consistent with other research in this field.

## Author Contributions

GB was a Ph.D. student under the supervision of GD. Both authors were involved in the conception of the idea behind this paper. GB collected and analyzed the data used in the paper, and wrote up the original paper, advised by GD. GD contributed to the writing of later drafts of the paper.

## Conflict of Interest Statement

The authors declare that the research was conducted in the absence of any commercial or financial relationships that could be construed as a potential conflict of interest.
